# Metastatic Carcinoma of Unknown Primary Presenting as Jugular Venous Thrombosis

**DOI:** 10.1155/2009/938907

**Published:** 2010-01-04

**Authors:** Prince Cheriyan Modayil, Sathyan Panthakalam, David C. Howlett

**Affiliations:** ^1^Department of Otorhinolaryngology, St George's Hospital, London SW17 0QT, UK; ^2^Department of Medicine, Eastbourne DGH, East Sussex BN21 2UD, UK; ^3^Department of Radiology, Eastbourne DGH, East Sussex BN21 2UD, UK

## Abstract

Jugular venous thrombosis is unusual and is associated with central venous catheterisation, intravenous drug abuse and head and neck sepsis. It is rarely associated with malignancy. We report a case of metastatic carcinoma of unknown primary in a forty year old female which presented with jugular venous thrombosis. The discussion includes investigation and treatment options for this condition.

## 1. Introduction

Historically jugular venous thrombosis has been associated with head and neck sepsis but has become increasingly uncommon since the introduction of antibiotics. According to Espritus and Media [1] central venous catheterisation and intravenous drug abuse are the most common aetiological factors recognised in current practice. Jugular venous thrombosis may also arise secondary to malignancy, and this association, although rare, should be considered in patients who present with this condition [2]. 

In this paper we describe a case of metastatic carcinoma of unknown primary which presented with a jugular venous thrombosis together with suggested investigation pathway and treatment options.

## 2. Case Report

A-40-year old female, previously fit and well, presented with pain and swelling of the left side of her neck of four days duration, in association with a sore throat and chest tightness. The pain radiated to the left axilla and shoulder. There was no history of fever or night sweats, and she had lost about 8 lbs in weight in the previous 3 months. She had no cough, and clinical history was otherwise unremarkable. She had no relevant past medical or surgical history and was not taking any medications. 

On examination, there was diffuse tender swelling on the left side of neck with marked engorgement of the superficial veins over this area, also involving the upper part of chest and upper left arm. There were a few scattered small tender lymph nodes in the posterior triangle of the left side of the neck. The remainder of clinical examination was normal. 

Initial laboratory investigations showed a normal full blood count, with a raised erythrocyte sedimentation rate of 58 mm and elevated C-reactive protein of 45 units (normal range is 0–10 units). Urea and electrolytes, liver function tests, and thyroid function tests were normal, and chest X-ray was also unremarkable. A thrombophilia screen was negative, and serum ANA was weakly positive. 

**Figure 1:**
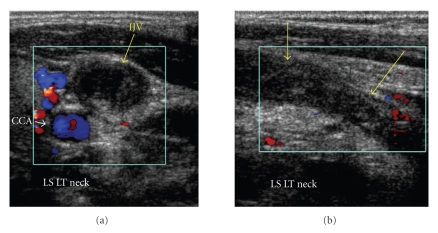
Transverse colour Doppler sonogram showing left internal jugular vein (IJV-arrow) containing thrombus and no colour flow. Note the normal colour flow in common carotid artery (CCA). Longitudinal colour Doppler sonogram showing thrombosed left IJV (arrows).

She was referred initially for ultrasound examination of the neck which demonstrated extensive thrombosis of the left internal and external jugular veins (Figures 1(a) and 1(b)).

**Figure 2:**
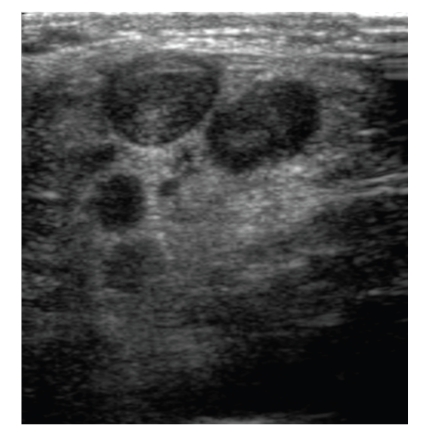
Sonogram of left supraclavicular fossa showing malignant left supraclavicular adenopathy.

Ultrasound also demonstrated enlarged lymph nodes in the left supraclavicular fossa which were suspicious of malignancy (Figure 2). 

**Figure 3:**
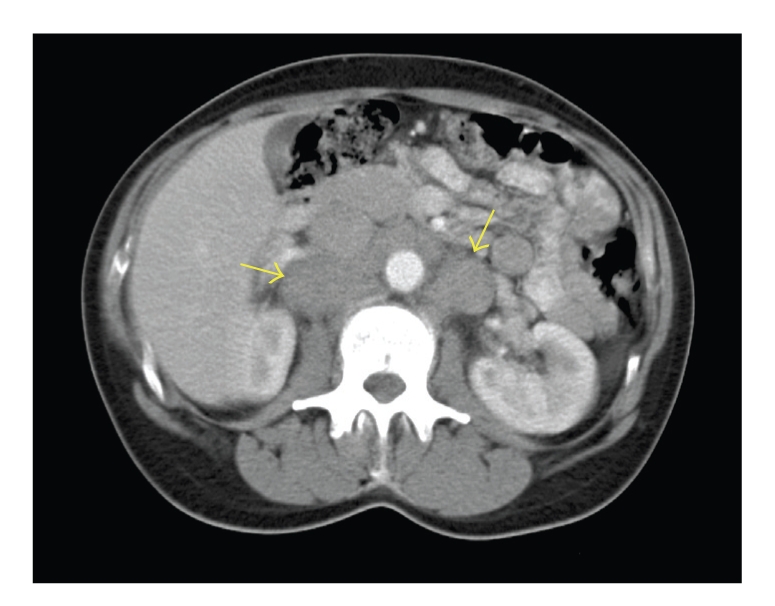
Axial postcontrast CT image at the level of the kidneys demonstrating extensive para-aortic lymphadenopathy (arrows).

She was initially treated with intravenous antibiotics and was anticoagulated with low molecular weight heparin followed by warfarin. Subsequently she underwent computed tomography (CT) of the neck, chest, abdomen, and pelvis which confirmed venous thrombosis of the left jugular veins. CT also showed multiple enlarged left supraclavicular nodes, an enlarged right retro-crural lymph node, and extensive portal and para-aortic adenopathy extending down to the aortic bifurcation (Figure 3). 

No other lesions were demonstrated, with the liver and lungs in particular appearing normal. 

This patient underwent an incisional biopsy of a left supraclavicular lymph node under general anaesthesia. The histology revealed replacement of normal node architecture by metastatic poorly differentiated carcinoma with probable squamoid differentiation. In order to find out the primary site she underwent panendoscopy of her upper aerodigestive tract which was normal. Further investigations failed to establish a possible primary site. This included gynaecological and pelvic examinations, proctosigmoidoscopy, tumour marker study, and CT scan of paranasal sinuses. A subsequent positron emission tomography also failed to identify a primary site malignancy. 

With a diagnosis of metastatic carcinoma of unknown primary, she was referred to the oncology department. She showed an initial response to chemotherapy and remained well at 6 months followup.

## 3. Discussion

Venous thrombosis usually affects the legs. The veins in the head and neck are less susceptible to thrombosis as they are mostly valveless and their drainage is aided by gravity in the upright position. 

Data relating to the natural history of jugular venous thrombosis is lacking. The prevalence rate for past or active malignancy as a risk factor for internal jugular venous thrombosis has been reported to be 29.7% [3]. Isolated jugular venous thrombosis is usually asymptomatic, whereas subclavian and axillary venous thrombosis produces pain and arm swelling [4]. 

The causes of jugular vein thrombosis may be local or systemic. Local factors include infections of paranasal sinuses, otological, facial, or orodental infections and local invasion by tumour encroaching the veins. Infective causes have been reduced significantly due to the use of antibiotics. In current everyday practice the more commonly recognised causes are central venous catheters, haemodialysis, and pacemakers. Of these, central venous catheter (CVC) associated thrombosis is the leading cause of jugular thrombosis. 

Systemic factors for jugular venous thrombosis include conditions that predispose to a hypercoagulable state [5]. These include thrombogenic disorders, myeloproliferative disorders, oestrogen use, pregnancy and the postpartum period, and malignancies. 

The incidence of venous thromboembolism is higher in cancer patients because of a combination of risk factors like host hypercoagulability, chemotherapy, immobilization, and insertion of CVCs. Long-term CVCs often facilitate chemotherapy, transfusions, parenteral nutrition, and blood sampling. However, vessel injury, venous stasis, and cancer related hypercoagulability increase the risk for CVC-related deep vein thrombosis. The incidence of clinically overt catheter associated upper-limb deep vein thrombosis has been reported to vary between 0.3% and 28.3% [6]. The incidence of clinically overt pulmonary embolism in such patients ranges from 15% to 25%.

The association between malignancy and thrombophlebitic events was first described by Armond Trousseau where migratory thrombophlebitis was associated with gastric carcinoma [7]. It is thought that tumour cells activate the blood coagulation system either by directly stimulating thrombin formation [8] or by inducing mononuclear cells to synthesize procoagulants such as tissue factors, prothrombin activators, and factor 5 activators. Cancer cells may also mediate platelet aggregation or activate endothelial cells by certain cytokines (TNF, IL-1) to generate substances that stimulate the production of coagulation tissue factors [9].

Pulmonary Embolism is a life threatening complication of jugular venous thrombosis and has been observed in 2.7% of cases [3]. Other complications include septic emboli, septicaemia, cerebral oedema, and pseudotumour cerebri [4].

The initial investigations should include full blood count, urea and electrolytes, liver function tests, clotting profile, thrombophilia, screen and tumour marker study.

Ultrasonography is an excellent diagnostic method for jugular venous thrombosis with an average sensitivity of 97 percent [10] and is the initial imaging modality of choice for delineation of venous thrombosis in both the deep veins of the leg and the neck. Venography is rarely used nowadays as it is invasive and involves contrast injection and exposure to radiation. Computed tomography and magnetic resonance venography have not yet been established. These modalities are however used to demonstrate adjacent structures in the neck, and to stage malignant disease. Although scanning describes the invasive properties of a specific mass, a biopsy often combined with upper aero digestive tract endoscopy is essential to establish a definitive diagnosis.

Treatment of jugular venous thrombosis includes anticoagulation with low molecular weight heparin followed by warfarin to prevent thromboembolic events. Superior venacaval filters are rarely indicated in isolated jugular venous thrombosis. However they can be used in patients with contraindication to anticoagulation. Other treatment modalities depend on individual cases and include use of antibiotics in the setting of infection, removal of central venous catheters, and appropriate chemo irradiation for malignant tumours. Jugular venous thrombosis due to deep neck infections may require drainage of fluid collections and local debridement. 

The treatment of CVC-associated thrombosis should be based on the prolonged use of low molecular weight heparin, except in the event of severe renal impairment when it should be based on the use of unfractionated heparin, rapidly replaced by warfarin [11]. Clinicians should be aware of the interaction between warfarin and 5-fluorouracil because of a high incidence of INR abnormalities and consequent bleeding risk [12].

Isolated case series discuss the use of thrombolytic drugs (streptokinase, urokinase, recombinant tissue plasminogen activator) in the treatment of CVC-associated thrombosis [13, 14]. Thrombolytic drugs are considered only in specific conditions where the thrombotic risk is higher than the risk associated with the use of these drugs, that is, in the event of vena cava syndrome [11]. 

There is insufficient evidence available on the value of catheter removal in CVC associated thrombosis. However, there is a real risk of embolisation during or immediately after catheter removal [1]. The catheter can be retained if it is mandatory, functional, in the right position and uninfected. In this case an anticoagulation treatment should be maintained as long as the catheter is retained [11]. The catheter removal is warranted in situations like malposition, infected thrombus, and irreversible occlusion of the lumen.

## 4. Conclusion

Jugular venous thrombosis may be first manifestation of an occult malignancy and may be due to the hyper coagulated state of the patient secondary to a metastatic malignancy. In the absence of an obvious head and neck pathology or haematological disorder, disseminated malignancy should be suspected and further investigations initiated.
